# Full‐body skin examination in screening for cutaneous malignancy: A focus on concealed sites and the practices of international dermatologists

**DOI:** 10.1002/jvc2.437

**Published:** 2024-04-29

**Authors:** James P. Pham, Nicholas Allen, Phoebe Star, Anne Cust, Pascale Guitera, Ashfaq A. Marghoob, John Paoli, Iris Zalaudek, Annika Smith

**Affiliations:** 1 School of Clinical Medicine, UNSW Medicine and Health, Sydney, New South Wales, Australia; 2 Faculty of Medicine and Health, The University of Sydney, Camperdown, New South Wales, Australia; 3 Melanoma Institute of Australia, North Sydney, New South Wales, Australia; 4 Sydney School of Public Health, The University of Sydney, Sydney, New South Wales, Australia; 5 Dermatology Service, Memorial Sloan Kettering Cancer Center, New York, USA; 6 Department of Dermatology and Venereology, Institute of Clinical Sciences, Sahlgrenska Academy, University of Gothenburg, Gothenburg, Sweden; 7 Department of Dermatology and Venereology, Region Västra Götaland, Sahlgrenska University Hospital, Gothenburg, Sweden; 8 Dermatology Clinic, Hospital Maggiore, University of Trieste, Trieste, Italy

**Keywords:** concealed site examination, full‐body skin examination, melanoma, nonmelanoma skin cancer, screening

## Abstract

**Background:**

Full‐body skin examination (FSE) is fundamental to the diagnosis of cutaneous malignancy but may not always include concealed site examination (CSE).

**Objectives:**

To determine the approach of international dermatologists to CSE during FSE and examine influencing factors, barriers and attitudes toward CSE.

**Methods:**

Members of the International Dermoscopy Society were surveyed using an online 12‐question survey disseminated via email.

**Results:**

There were 706 completed responses among 1249 unique clicks to the survey, representing a completion rate of 56.5%. Fifty‐four percent of respondents reported always examining the breasts, while 52.8%, 18.8%, and 11.8% always examined the scalp, oral, and anogenital mucosa, respectively. The most frequent reason for examining concealed sites was patient concern, whilst common reasons for not examining concealed sites included low incidence of pathology and concern regarding allegations of sexual misconduct.

**Conclusions:**

Our findings allude to the need for international consensus guidelines regarding the conduct and inclusion of concealed or sensitive sites in routine FSE. This is essential to define clinician responsibilities, inform patient expectations of care, and thereby mitigate potential medicolegal repercussions.

## INTRODUCTION

The incidence of melanoma and nonmelanoma skin cancers (NMSCs) appear to be increasing globally.^1^ Clinicians rely on full‐body skin examination (FSE) as the mainstay of screening for and clinical detection of melanoma and NMSCs.^2^ The process of FSE is often suggested to entail a total examination of the skin, that is inspection of all skin including mucous membranes^3^; however practice may vary between examiners.^4^

FSE is a valuable clinical adjunct allowing for detection of melanomas with shallower Breslow depths, lower likelihood of metastatic disease and therefore better prognosis.^5,6^ However, some anatomic locations are not readily observable including the scalp, breasts, anogenitalia and oral mucosae. While these concealed sites have a lower incidence of malignancy, delay in seeking medical assessment at these sites may correlate with more advanced disease and thus poorer prognosis.^7–12^ Barriers to concealed site examination (CSE) may include patient sensitivity to their inspection, clinician time constraints or lack of available chaperones.^13–16^

Studies evaluating the practice of FSE including the examination of concealed sites have pointed to a lack of accepted standard of practice.^4,17^ A consensus approach to FSE and CSE serves to provide clinicians with clear guidance as to expectations of conduct when performing these examinations. Similarly, a public framework could inform patient expectations of care and potentially minimise some of the difficulties clinicians face in approaching CSE, particularly with regard to potentially sensitive sites such as the breasts (in women) and anogenitalia.

This study seeks to lay the groundwork for an international consensus approach to the inclusion of CSE as part of the FSE by establishing what constitutes current practices, influencing factors, barriers and attitudes concerning practice across the globe. These results will also be compared to the findings of an Australian based survey‐study assessing practice amongst Australian Dermatologists practicing in the world’s epicentre of skin cancer.^18^

## METHODS

An invitation to participate in an anonymous 11‐question online survey was disseminated via email to members of The International Dermoscopy Society (IDS) in 2021, following development and review by a panel of experts. The survey was utilised in two studies across two cohorts, one pertaining to Australian dermatologists (members of the ACD)^18^ to capture a national perspective, noting the higher prevalence of both melanoma and NMSC in Australia, and the second study to an international cohort of dermatologists. Participants gave implied consent by completion of the survey, and only Consultant Dermatologists were requested to complete surveys. Two reminder emails were disseminated 2 weeks apart following the initial invitation. The survey was closed after 6 weeks.

Data was collected to establish the demographic features of respondents and their approaches to and attitudes regarding FSE with a focus on concealed sites, namely the scalp, breasts, oral and anogenital mucosae. Other details collected from respondents included factors influencing their decision‐making regarding CSE, chaperone use and whether they felt dermatologists should be responsible for examining concealed sites as part of the FSE. The full survey questions distributed are provided in Table S1.

Primary outcomes were clinician‐reported frequency, practice and attitudes regarding the inclusion of concealed sites in the FSE. Descriptive statistics regarding responses to each question were extracted from Google Survey Forms. Statistical comparisons between Australian and International survey respondent characteristics and practice were made using nonparametric tests given the non‐Gaussian nature of the data, with *p* < 0.05 considered significant. All data analysis was conducted using Graphpad Prism v9.4.1.

Ethics approval was obtained from St Vincent’s Human Research Ethics Committee (2019/ETH12379).

## RESULTS

From 1249 unique clicks on the email, there were 706 responses—representing a completion rate of 56.5% for international dermatologists, compared to 45.9% for the ACD survey (Table 1). Compared to the Australian cohort, more of the international respondents were female (64.4% vs. 51.9%, *p* < 0.001) and a greater proportion was 35 years old or younger (18.7% vs. 8.4%, *p* < 0.001); while fewer report FSE comprising over half of their patient cohort (39.8% vs. 49.7%, *p* < 0.001).

**FIGURE 1:**
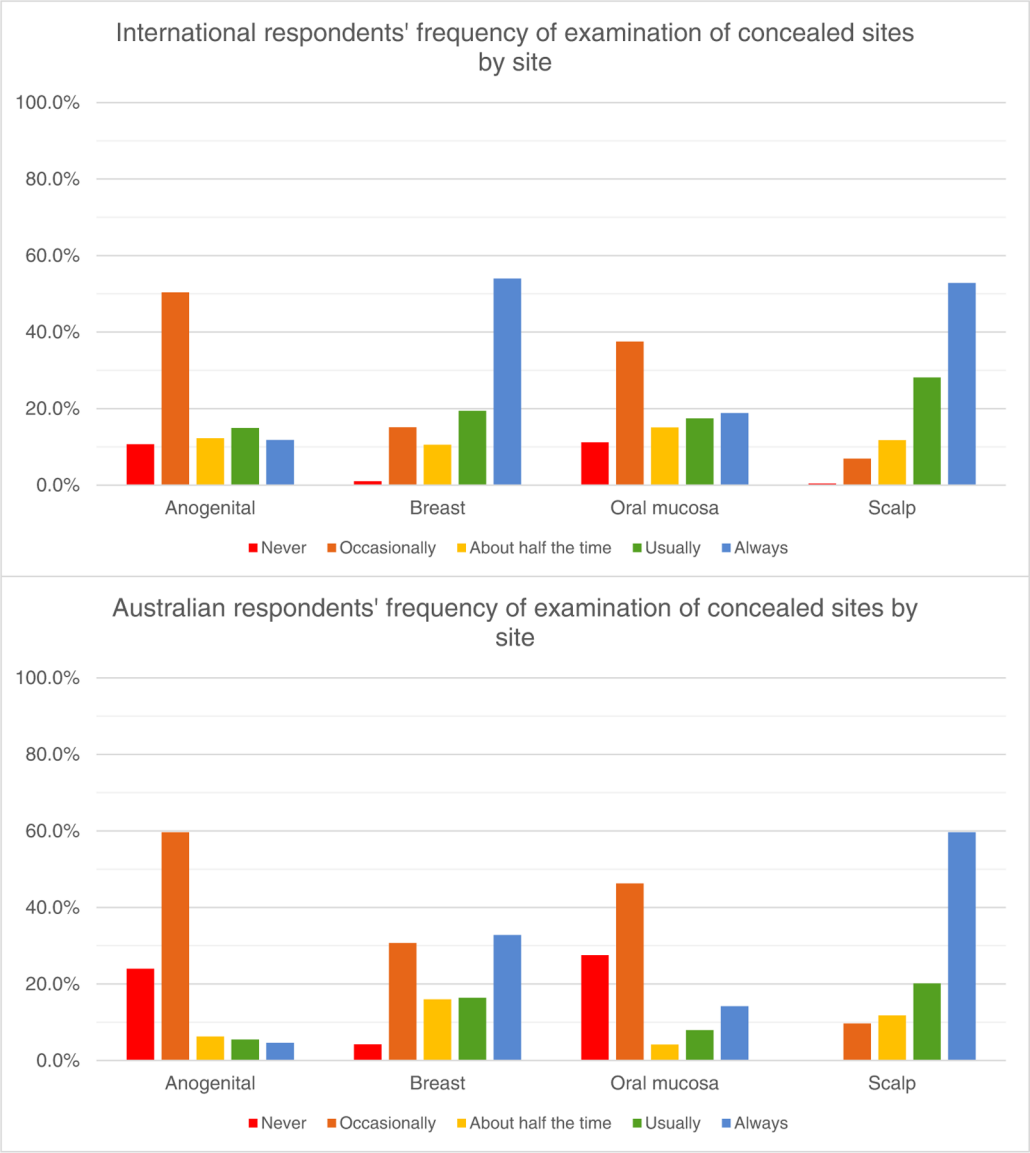
Respondents’ frequency of examination of concealed sites by site.

The frequency with which respondents reported examining concealed sites as part of the FSE is presented in Figure 1. Most respondents in both international and Australian cohorts reported always examining the scalp (52.8% vs. 59.4%, respectively, *p* = 0.07), however international respondents more often reported routinely examining the breasts (54.0% vs. 32.9%, *p* < 0.001), oral mucosa (18.8% vs. 14.3%, *p* = 0.11) and anogenitalia (11.8% vs. 3.6%, *p* < 0.001).

Regarding reasons prompting clinician examination of concealed sites, both international and Australian respondents favoured patient concern when considering whether to examine the oral mucosa (46.0% vs. 64.1%, respectively, *p* < 0.001) and anogenital mucosa (32.6% vs. 80.6%, *p* < 0.001) as the predominant justification (Figure S1). Amongst both international and Australian dermatologists, examination of the scalp and breasts was most likely to be reported as in accordance with ‘best practice’ (61.1% vs. 53.6%, *p* = 0.04). Free text responses indicated many tailored their approach to FSE based on individual patient factors, for example a history of malignancy at concealed sites or risk factors such as lichen sclerosus for vulval carcinoma.

When deciding *not* to examine the oral mucosa, both groups of respondents justified this with the low incidence of pathology at this site (international 32.6%, Australian 40.1%, *p* = 0.03) (Figure S2). Notably, the international survey was disseminated in 2021 at the height of the COVID‐19 pandemic (compared to 2018 for the Australian survey), and thus many dermatologists indicated via free text response that they avoided examining sensitive sites, namely oral sites to reduce infectious transmission risk and consult time. With regard to the anogenital area, more Australian dermatologists reported concerns regarding potential accusations of sexual misconduct (26.3% vs. 43.5%, *p* < 0.001) and low incidence of pathology (25.5% vs. 44.7%, *p* < 0.001). Similar to their Australian counterparts, many international dermatologists reported offering examination of the anogenital area according to patient concern (international 55.7% vs. Australian 63.3%, *p* = 0.04) (Figure S1), while routinely examining the scalp (77.0%) and breasts (62.8%).

With respect to risks of *not* routinely examining concealed sites, the Australian and international cohorts expressed similar concerns about the possibility of missed diagnosis of cutaneous malignancy (international 90.9% vs. Australian 94.1%, *p* = 0.013), the fear of medical negligence (international 51.0% vs. Australian 65.0%, *p* < 0.001), patient perception of examination thoroughness or lack thereof (international 37.3% vs. Australian 48.5%, *p* < 0.001) and outsourcing diagnostic responsibility to other specialists (international 22.0% vs. Australian 27.0%, *p* = 0.11). Respondents also frequently reported perceived barriers to CSE being patient embarrassment (international 83.0% vs. Australian 90.3%, *p* = 0.007), patients’ lack of skin cancer knowledge (international 54.8% vs. Australian 54.0%, *p* = 0.83), and patient preference for clinician gender (international 41.1% vs. Australian 58.2%, *p* < 0.001). Concerns raised by international dermatologists via free text response included the potential for CSE to act as a deterrent for patients to attend future FSE, due to embarrassment or shame; and increasing time constraints of FSE.

International respondents provided similar answers to Australian dermatologists regarding recruitment of chaperones, with 36.3% stating that they never recruit a chaperone when examining anogenital areas (vs. Australian 39.3%, *p* = 0.41) and 52.8% recruiting a chaperone for examination of the breasts (vs. Australian 61.1%, *p* = 0.025). Both international and Australian dermatologists reported using chaperones in settings involving young patients (international 46.5% vs. Australian 43.5%, *p* = 0.42); where respondents felt uncomfortable with the patient or the examination (42.8% vs. 45.6%, *p* = 0.44); by patient request (42.5% vs. 46.4%, *p* = 0.29); or with female patients (33.6% vs. 45.1%, *p* = 0.01).

In contrast to International respondents, fewer Australian dermatologists (74.5% vs. 40.5%, respectively, *p* < 0.001) believed examination of concealed sites falls within expected scope of practice of dermatologists. By site, more international respondents believed dermatologists are also responsible for examining oral and anogenital mucosa (31.6% and 32.1%, respectively), compared to the Australian cohort (oral mucosa 19.0%, anogenitalia 20.3%; *p* < 0.001 for both). Free text responses in the international survey suggested greater involvement of other specialists such as dentists, gynaecologists and urologists in examining concealed sites for routine health screening, rather than dermatologists, which was not evident in the Australian survey.

## DISCUSSION

Most international survey respondents felt it was their duty to examine concealed sites, which contrasts with the more divided opinions of the Australian cohort—74.5% vs. 40.5%, respectively. By site, most dermatologists in both cohorts agree that examination of the scalp and breast falls within expected scope of practice as part of routine conduct FSE. Moreover, international respondents were significantly more accepting of the responsibility to examine the oral mucosa and anogenital region (31.5 and 32.2% respectively) compared to Australian counterparts (19.0% and 20.3%), although absolute proportions remain low. As such, we propose patients be educated and informed to routinely self‐survey concealed sites as part of their routine skin self‐surveillance and nominate lesions of concern in concealed sites for review by their doctor at the time of formal FSE.

The incidence of malignancies such as melanoma and NMSCs in concealed sites should not be discounted. For instance, vulvar melanomas account for 3−7% of melanoma diagnoses in women overall and are associated with high mortality rates of up to 70%, hypothesised to be due to later stage at diagnosis.^19^ Similarly, scalp melanomas, despite comprising only 1−2% of melanoma diagnoses, are associated with a sixfold increase in mortality risk compared to cutaneous melanoma arising at other body sites, even following adjustment for Breslow thickness.^20^

Given the disparity between the lower incidence of and poorer outcomes associated with skin cancers arising at concealed sites, it is important to establish consensus as to whether routine FSE should entail inclusion of concealed sites. This is reflected by the breadth of clinician responses in the international and Australian surveys, highlighting the need for a standardised approach. Ideally, clear evidence would be available that would support a particular method of FSE, with regard to cost–benefit analyses and assessment of patient centred outcomes such as melanoma or NMSC‐specific mortality. What is more likely is that a complex collection of evidence of different methodologies will need to be synthesised and interpreted leading to the development of guidelines that are both evidence and consensus‐based.^21^

Routine inclusion of breast examination in the FSE by most dermatologists has been previously described in a study surveying American dermatologists in high‐risk skin cancer clinics.^4^ A survey of Canadian dermatologists found a similar proportion include the breasts in their FSE—34.5% compared to 54.0% and 32.9% for International and Australian dermatologists in our studies, respectively.^17^ However, there was a difference between the practice of male and female dermatologists, with female clinicians more likely to examine the breasts (45.4 vs. 18.2%, *p* = 0.04). However, such cross‐sectional studies focus on dermatologists working with high‐risk patient groups rather than a general FSE cohort as assessed in our study.^4,17^

Clinicians appear to examine concealed sites more frequently in high risk cohorts,^4^ consistent with free text responses proposing patient tailored approached based on patient/clinician concern and individual patient risk factors for malignancy at these sites. For example, a recent meta‐analysis has shown male androgenetic alopecia increases the risk of head and neck melanoma by 31%, whereas risk for keratinocyte cancers was not increased.^22^ Further, Bishop et al. found that melanomas arising at mucosal sites, including the oral cavity, external genitalia and rectum/anus, were more likely to be nodular and diagnosed at a more advanced stage.^23^ It should be noted that in the advanced or metastatic setting, mucosal melanomas respond poorly to systemic therapies such as immune checkpoint inhibition compared to cutaneous melanoma—highlighting risks of delayed diagnosis.^24^ Notably, the proportion of which mucosal melanomas constitute overall melanoma diagnoses vary between ethnic populations; from 1% in White patients to 8−25% in Black or Asian groups,^25^ suggesting best practice regarding CSE may warrant ethnicity‐specific considerations.

As noted in free text responses, dermatologists may attribute responsibility for examining the oral mucosa to dentists and oral medicine clinicians who may examine these sites more routinely and with appropriate equipment, or general practitioners and gynaecologists who conduct Pap smear examinations for women. Such practices may vary internationally, noting that in Australia most melanoma diagnoses are made by General Practitioners,^26,27^ who may be similarly equipped for skin cancer screening at sensitive sites. A parallel may be drawn to the screening of ocular melanoma, which is principally diagnosed by ophthalmologists after the development of lesions of concern (as noticed by the patient or other clinicians) rather than routine screening.^28^ Nevertheless, this points to a potential role that dermatology bodies, such as the IDS and ACD, may have in equipping general practitioners, gynaecologists, dentists and even hairdressers (with respect to the scalp) with the necessary skills needed to inspect these sites and refer to dermatologists as appropriate for lesions that warrant further investigation.

A limitation of this study is the overrepresentation of European dermatologists, meaning the results may not represent practices in other regions internationally. Moreover, there was a small contingent of international dermatologists who reported practicing in Australia or Oceania. If these dermatologists were members of the ACD, they may have been surveyed twice in the IDS and ACD surveys, although the surveys requested completion only once. In addition, while only Consultant Dermatologists were requested to complete the survey, we acknowledge the potential for other practitioners (such as General Practitioners) who may have erroneously submitted responses which we were unable to identify or account for. As only cohort‐level data were collected in our surveys, we are unable to identify predictive factors toward CSE inclusion in FSE such as clinician sex, age or country of practice. Further, self‐report as a method of data collection is retrospective and leaves room for inaccurate responses. However, we feel that given how frequently FSE is performed by dermatologists, the potential for recall bias should be negligible.

Further work is required to establish a standard approach to FSE, concerning the need for routine inclusion of CSE, to inform dermatologists of their clinical responsibilities, the expectations of their practice and set appropriate patient expectations for care. Investigations that may better assist development of consensus guidelines on routine integration of CSE in FSE include evaluating the merit of CSE in high‐risk patient cohorts with respect to mortality benefit, cost‐utility and patient acceptability, and the clinical and biologic behaviour of melanomas and NMSCs arising in concealed anatomical sites. Ideally, this should incorporate approaches aiming to improve patient awareness of cutaneous malignancy risk at concealed sites, for example information leaflets.^29^ Such measures may encourage self‐examination and allow for targeted evaluation of new or suspicious lesions during FSE visits with dermatologists, rather than a blanket approach to screening all patients.

## CONCLUSION

**FIGURE 2:**
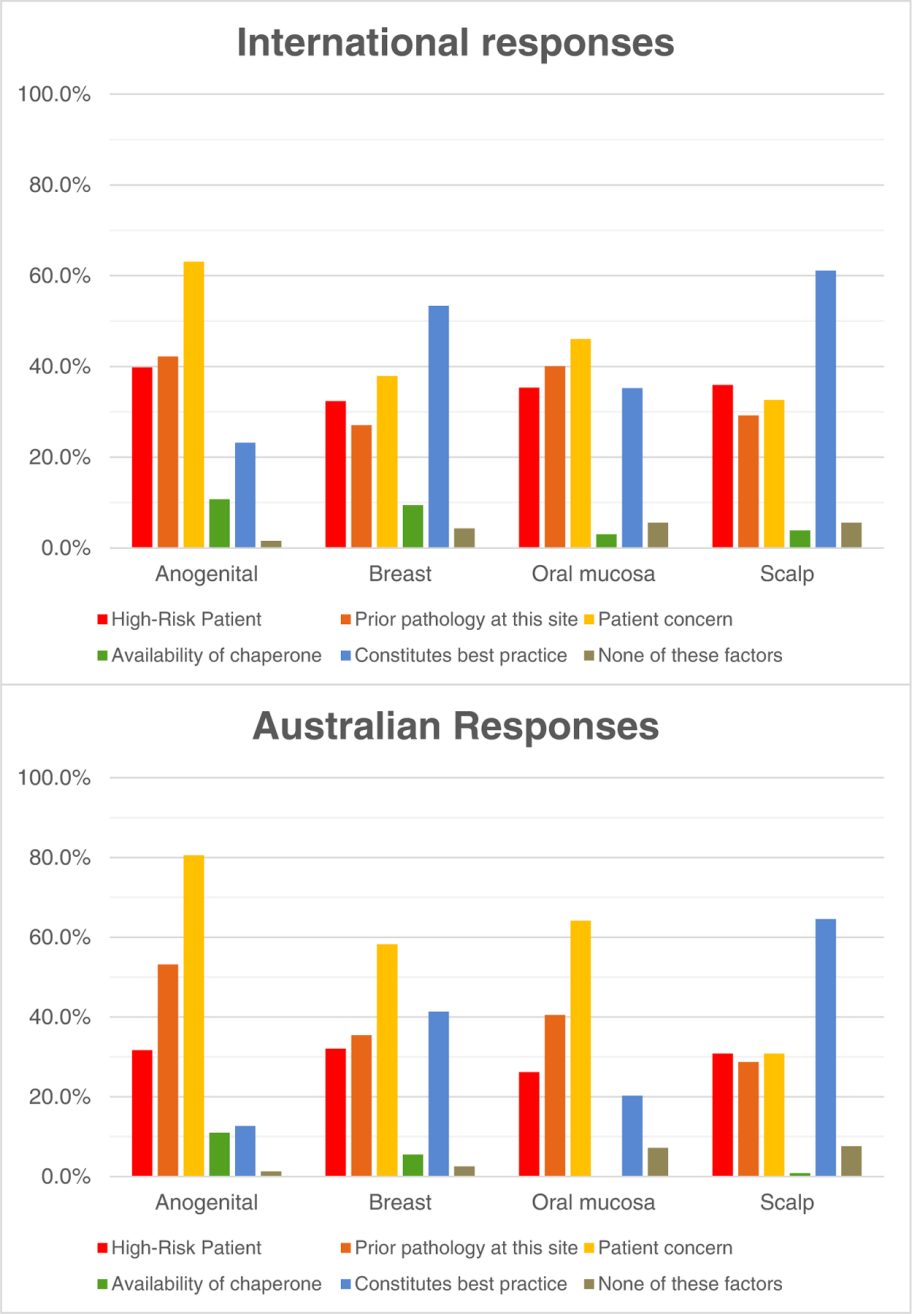
Factors that influence the decision to examine concealed sites by site.

Based on combined Australian and international dermatologist responses, we propose that FSE routinely include examination of the scalp, while inclusion of breast and anogenital or oral mucosal sites should be guided by patient concern, individual risk factors and clinician discretion. Establishment of an evidence based consensus approach is needed to support these findings. Furthermore, a consensus‐based approach toward patient education regarding the risk of cutaneous malignancies arising at concealed sites and the importance of self‐examination is necessary to reduce risk of delayed diagnosis and its associated morbidity (Figure 2).

## Supplementary Material

Figurementary Figure 1: Factors that influence the decision not to examine concealed sites by site.

Supporting Information.

Figurementary Figure 2: Respondents' practices regarding the practice of offering examination of concealed sites by site.

## Figures and Tables

**TABLE 1:** Description of respondents.

Characteristic	International respondents (*N* = 706)	Australian respondents (*N* = 237)
*Gender*		
Female	455 (64.4%)	123 (51.9%)
Male	251 (35.6%)	114 (48.1%)
*Age group*		
35 years old or younger	132 (18.7%)	20 (8.4%)
36−45 years	198 (28.0%)	63 (26.6%)
46−55 years	169 (23.9%)	64 (27.0%)
Older than 55 years	207 (29.3%)	90 (38.0%)
*Location of practice*		
Europe	396 (56.1%)	‐
North America	64 (9.1%)	‐
South America	60 (8.5%)	‐
Asia	35 (4.9%)	‐
Africa	18 (2.6%)	‐
Other	56 (7.9%)	‐
Australia/Oceania	77 (10.9%)	237 (100%)
*Proportion of patient cohort who are offered a full*‐*body skin examination*		
≤25%	236 (33.4%)	34 (14.3%)
26−50%	189 (26.8%)	85 (35.9%)
51−75%	130 (18.4%)	85 (35.9%)
76−100%	151 (21.4%)	33 (13.9%)

## Data Availability

The data that support the findings of this study are available from the corresponding author, (A. S.) upon reasonable request.
