# Supplementary material for: Full‐body skin examination in screening for cutaneous malignancy: A focus on concealed sites and the practices of international dermatologists
Source: JEADV Clin Pract. Author manuscript; Available in PMC 2025 Oct 9. (PMC12507240; doi:10.1002/jvc2.437)
Supplement: Figurementary Figure 1: Factors that influence the decision not to examine concealed sites by site. [file NIHMS2045300-supplement-Figurementary_Figure_1.pdf]

- What is your gender?
    - Male
    - Female
- What is your age group?
    - ≤ 35
    - 36-45
    - 46-55
    - ≥ 55
- What percentage of your total patient cohort present for full-body skin examination (FSE)?
    - ≤ 25%
    - 26-50%
    - 51-75%
    - ≥ 76%
- How often do you examine the following sites are part of your FSE? (*subcategories included for anogenital, breasts, oral mucosa and scalp)*
    - Always
    - Usually
    - About half the time
    - Occasionally
    - Never
- Which of these factors influence your decision TO examine the following sites as part of your FSE? (*subcategories included for anogenital, breasts, oral mucosa and scalp)*

    - High risk patient
    - Prior pathology at the site
    - Patient concern
    - Availability of chaperone
    - *Free text responses*
- Which of these factors influence your decision to NOT examine the following sites as part of your FSE? (*subcategories included for anogenital, breasts, oral mucosa and scalp)*
    - Not required as part of FSE
    - Lack of time
    - Low incidence of pathology
    - Chaperone unavailable
    - *Free text responses*

- When performing FSE for the following sites (*subcategories included for anogenital, breasts, oral mucosa and scalp*) I do the following:
    - Examine routinely
    - Offer examination according to patient risk factors
    - Examine only at patient request
    - Partly examine only
    - *Free text responses*
-
    - How often do you recruit the help of a chaperone to assist with FSE of the following sites? (*subcategories included for anogenital and*

breasts)

    - Always
    - Usually
    - About half the time
    - Occasionally
    - Never

  - In what setting are you more likely to recruit a chaperone for concealed site examination? (*subcategories included for anogenital and breasts*)
    - Male patient
    - Female patient
    - Younger patient
    - Older patient
    - Physician of different gender
    - Only if patient requests
    - *Free text responses*

- What factors have you observed that may serve as barriers to patients accepting inclusion of concealed sites (anogenital, breasts, oral mucosa, scalp) in routine FSE?
  - Patient embarrassment
  - Patient physician gender preference
  - Patient preference for GP or other practitioners to examine these sites
  - Lack of patient knowledge regarding possibility of malignancy at these sites

    - *Free text responses*

- What issues do you foresee could potentially arise in NOT including examination of concealed sites (anogenital, breasts, oral mucosa, scalp) in the FSE?
    - Missed diagnosis of cutaneous malignancy
    - Risk of medical negligence
    - Patient perception of lack of thoroughness of examination
    - Outsourcing diagnostic responsibility to other specialists
    - *Free text responses*

-

  - Do you agree it is the Dermatologist’s responsibility to routinely include examination of the following sites (*subcategories included for anogenital, breasts, oral mucosa and scalp*) as part of screening for cutaneous malignancy in FSE?
    - Yes
    - No
    - Unsure
  - If your answer to 11a) is site dependent please indicate below (Optional)
    - Anogenital – yes/no/unsure
    - Breasts – yes/no/unsure
    - Oral mucosa – yes/no/unsure
    - Scalp – yes/no/unsure
