# Supplementary figures and images for: Full‐body skin examination in screening for cutaneous malignancy: A focus on concealed sites and the practices of international dermatologists
Source: JEADV Clin Pract. Author manuscript; Available in PMC 2025 Oct 9. (PMC12507240; doi:10.1002/jvc2.437)

## International responses

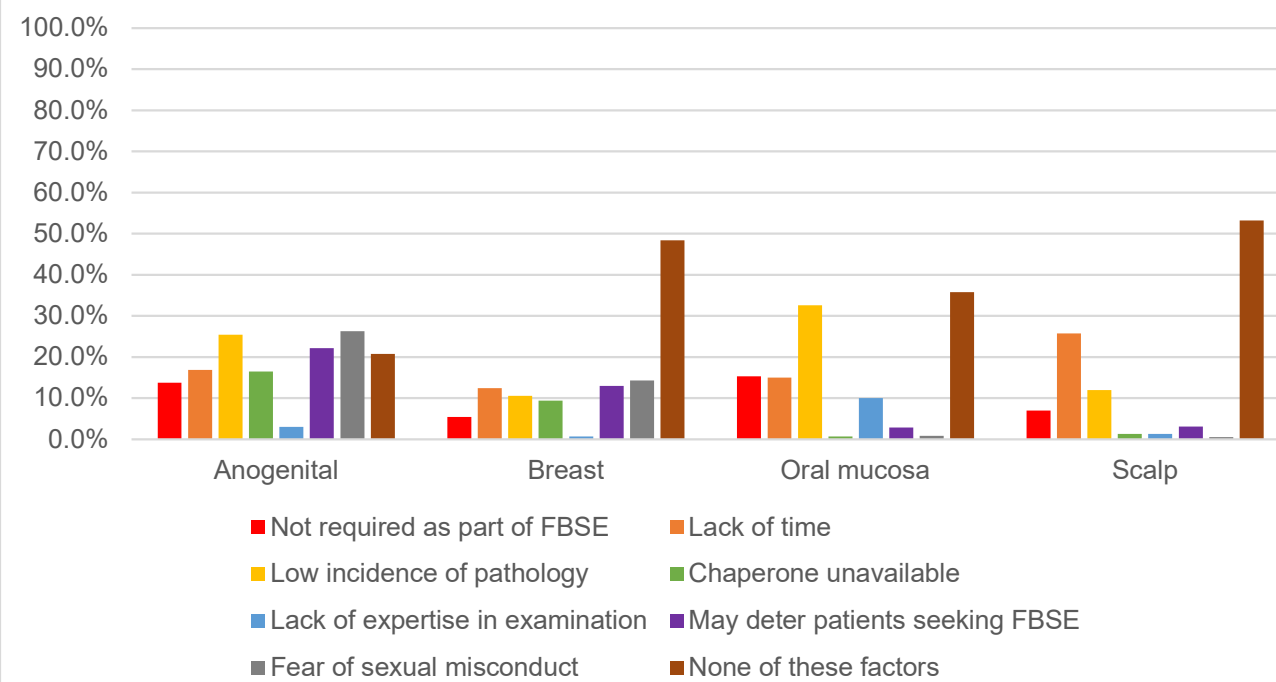

## Australian responses

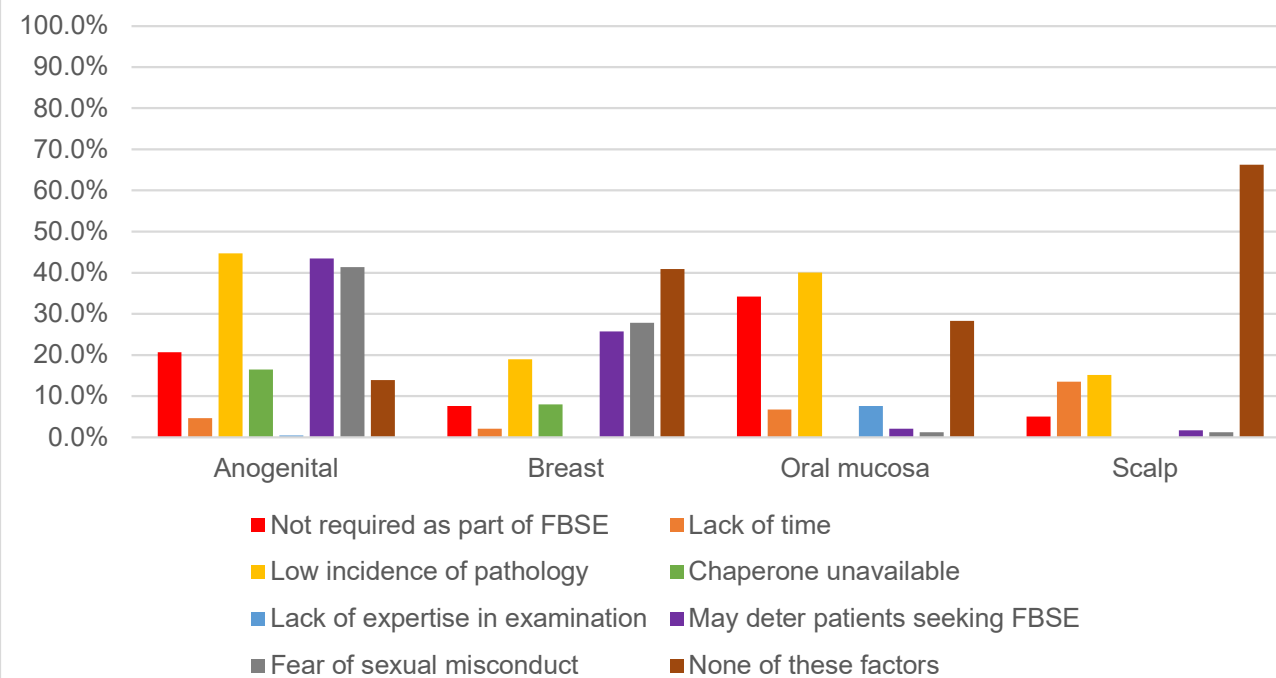

Supplement: Supporting Information. [file NIHMS2045300-supplement-Supporting_Information_.pdf]

## International Responses

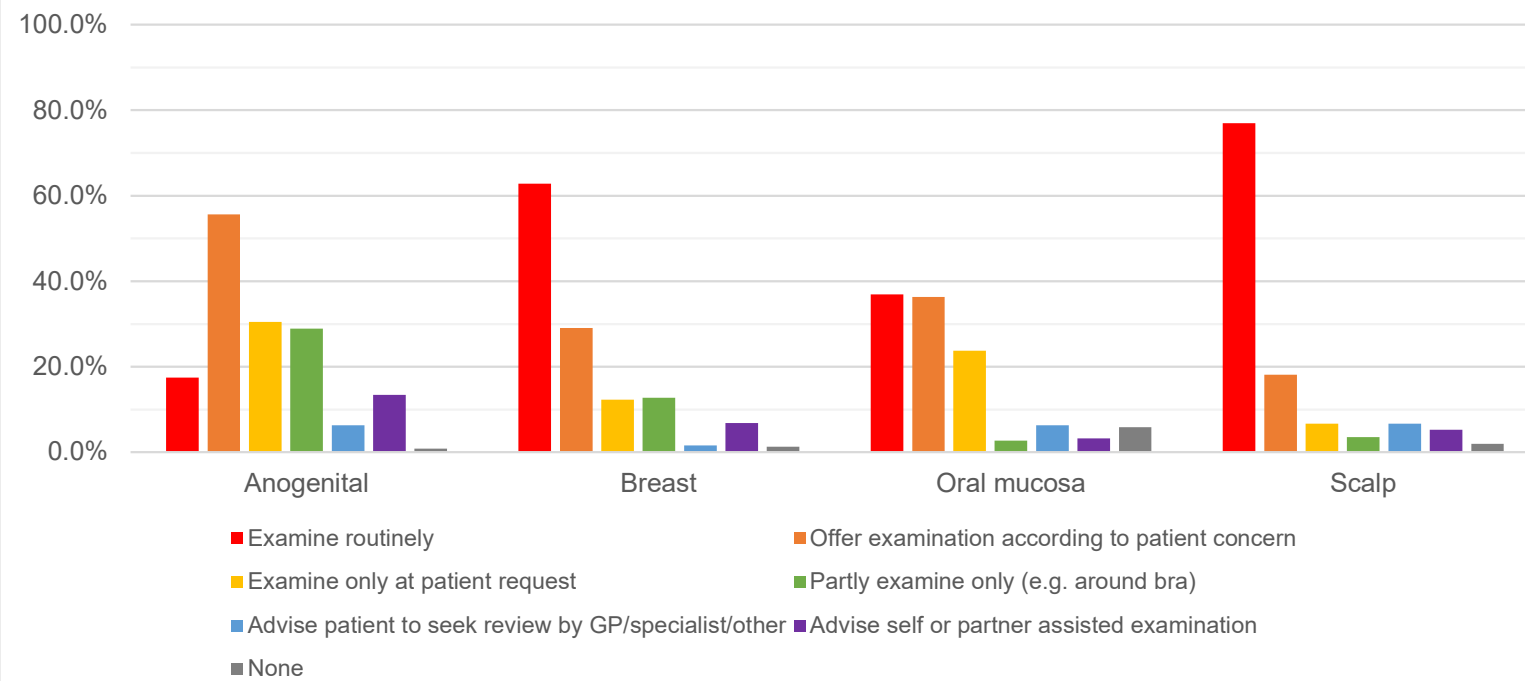

## Australian Responses

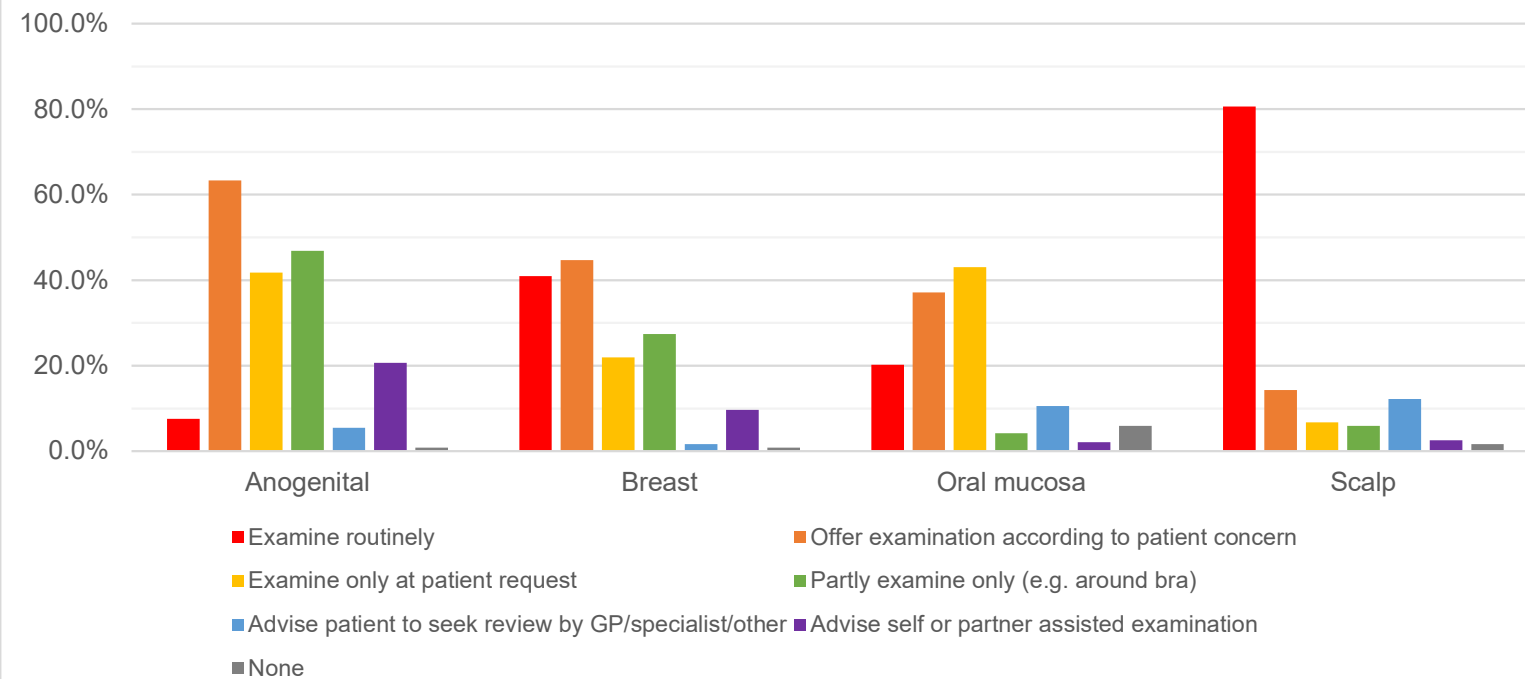

Supplement: Figurementary Figure 2: Respondents' practices regarding the practice of offering examination of concealed sites by site. [file NIHMS2045300-supplement-Figurementary_Figure_2.pdf]
